# Mailed HPV self-sampling for cervical cancer screening among underserved minority women: study protocol for a randomized controlled trial

**DOI:** 10.1186/s13063-016-1721-6

**Published:** 2017-01-13

**Authors:** Erin Kobetz, Julia Seay, Anthony Amofah, Larry Pierre, Jordan Baeker Bispo, Dinah Trevil, Martha Gonzalez, Martine Poitevien, Tulay Koru-Sengul, Olveen Carrasquillo

**Affiliations:** 1 Sylvester Comprehensive Cancer Center, University of Miami, Miller School of Medicine, Clinical Research Building, 1120 NW 14th Street, Room 610B, Miami, FL 33136 USA; 2 Department of Medicine, University of Miami Miller School of Medicine, 1120 NW 14th Street, Miami, FL 33136 USA; 3 Health Choice Network, 9064 NW 13th Terrace, Miami, FL 33172 USA; 4 Center for Haitian Studies, 8260 NE 2nd Avenue, Miami, FL 33138 USA; 5 Department of Public Health Sciences, University of Miami Miller School of Medicine, 1120 NW 14th Street, Miami, FL 33136 USA

**Keywords:** Cervical cancer, HPV, Haitian, Hispanic, Immigrant, Screening

## Abstract

**Background:**

Underserved ethnic minority women experience significant disparities in cervical cancer incidence and mortality, mainly due to lack of cervical cancer screening. Barriers to Pap smear screening include lack of knowledge, lack of health insurance and access, and cultural beliefs regarding disease prevention. In our previous SUCCESS trial, we demonstrated that HPV self-sampling delivered by a community health worker (CHW) is efficacious in circumventing these barriers. This approach increased screening uptake relative to navigation to Pap smear screening. SUCCESS trial participants, as well as our community partners, provided feedback that women may prefer the HPV self-sampler to be delivered through the mail, such that they would not need to schedule an appointment with the CHW. Thus, our current trial aims to elucidate the efficacy of the HPV self-sampling method when delivered via mail.

**Design:**

We are conducting a randomized controlled trial among 600 Haitian, Hispanic, and African-American women from the South Florida communities of Little Haiti, Hialeah, and South Dade. Women between the ages of 30 and 65 years who have not had a Pap smear within the past 3 years are eligible for the study. Women are recruited by CHWs and complete a structured interview to assess multilevel determinants of cervical cancer risk. Women are then randomized to receive HPV self-sampling delivered by either the CHW (group 1) or via mail (group 2). The primary outcome is completion of HPV self-sampling within 6 months post enrollment.

**Discussion:**

Our trial is among the first to examine the efficacy of the mailed HPV self-sampling approach. If found to be efficacious, this approach may represent a cost-effective strategy for cervical cancer screening within underserved and underscreened minority groups.

**Trial registration:**

ClinicalTrials.gov, NCT02202109. Registered on 9 July 2014.

**Electronic supplementary material:**

The online version of this article (doi:10.1186/s13063-016-1721-6) contains supplementary material, which is available to authorized users.

## Background

Widespread adoption of the Pap smear has significantly reduced cervical cancer incidence and mortality in the US [1]. However, not all US women have benefited equally. Minority, low-income, and underinsured women remain at excess risk of being diagnosed with and dying from cervical cancer [2–4]. This disparity is particularly problematic in light of the enormous financial, quality of life, and emotional burden associated with cervical cancer management. In 2010, the estimated annual net cost of cervical cancer treatment during the initial treatment phase was projected to be US$54,209 for women aged under 65 years and US$45,174 for women aged 65 years and older [5]. Quality of life in cervical cancer patients is often impacted by increased depression, anxiety, fear, distortion of body image, diminished self-perceived femininity and self-esteem, and sexual and reproductive issues [6]. In Miami, FL, cervical cancer is a particular problem for Haitian and Caribbean Hispanic women. When compared to other racial/ethnic minority and immigrant groups in the Miami metropolitan area, Haitian and Hispanic women contribute disproportionately to cervical cancer incidence, morbidity and mortality, largely as a function of their underutilization of Pap smear screening [7–10]. They do not receive routine Pap smears for myriad reasons including but not limited to: poverty, language difficulties, limited access to health care, lack of knowledge about cancer and the importance of early detection, cancer fatalism or the belief that cancer implies death, and cultural norms about health and about disease prevention [7, 11–18].

Alternative screening strategies, such as human papilloma virus (HPV) self-sampling, may circumvent the aforementioned barriers to cervical cancer screening within these groups [19]. Briefly, HPV self-sampling enables women to test themselves for HPV outside a clinical setting, and has consistently been shown to be as sensitive as physician-collected specimens for HPV detection [20]. HPV testing examines the presence of high-risk HPV, the primary cause of cervical cancer, while Pap smear screening examines the presence of precancerous cell abnormalities. While not the current standard-of-care for cervical cancer screening, HPV testing has demonstrated superior sensitivity and negative predictive value when compared with Pap smear screening [21]. In other countries, randomized studies of HPV self-sampling have shown increased rates of screening [19, 22]. In the US, nonrandomized studies have also shown that this approach has high acceptability among minority women [19, 23].

We have recently completed a randomized controlled trial of community health worker (CHW)-delivered, home-based HPV self-sampling among Haitian and Hispanic immigrant women living in South Florida, through our National Cancer Institute (NCI)-funded South Florida Center for Reducing Cancer Disparities (SUCCESS) [24]. When compared with both standard educational outreach, as well as CHW-guided navigation to Pap smear screening, HPV self-sampling was superior in increasing cervical cancer screening uptake. Given that HPV self-sampling has been shown to be the superior method of cervical cancer screening within our target communities, our focus has shifted to understanding how best to implement self-sampler delivery. Participant feedback from our SUCCESS trial indicated a possible preference for a mailed approach, which would eliminate the need for a CHW to visit participants’ homes to deliver the self-sampler. Given our results, as well as our participant feedback, for the current trial we are examining the efficacy of a mailed HPV self-sampling approach versus home-based HPV self-sampling delivery in increasing cervical cancer screening uptake among immigrant women living in South Florida.

### Aims and objectives

As in our prior trial, our first aim is to examine uptake of cervical cancer screening by women assigned to HPV self-sampling via mail versus home-based HPV self-sampling delivered by a CHW. Our second aim is to examine the efficacy of these interventions by site, ethnic group, and acculturation level. Finally, we aim to examine the impact of these interventions on secondary outcomes including knowledge, attitudes, and beliefs about cervical cancer and the importance of early detection of disease and access to care, as well as to determine the relative cost of the two intervention strategies. Ultimately, our trial will determine the optimal strategy for delivering the HPV self-sampling intervention.

## Methods/Design

### Trial design/setting

We are recruiting 600 women from three South Florida immigrant communities: Little Haiti, Hialeah, and South Dade. Minority women aged 30–65 years who have not undergone cervical cancer screening in the past 3 years are recruited to participate by a CHW, based at a partner organization in their community of residence. Women who agree to participate will have a 30-min interview in their home by one of our two bilingual Community Health Educators (CHEs). Upon completion of the interview, participants are randomized into one of two interventions. The CHW self-sampling intervention is our control arm (CHW + SS). Women randomized to this arm receive a home visit by a CHW, who provides education about cervical cancer and the importance of early detection of disease, as well as detailed instruction for how to self-sample for HPV. In comparison, women in our experimental arm (SS + M) receive the self-sampler by mail along with print material that provides information about cervical cancer and instructions for using the self-sampler. The study procedure timeline is outlined in Table1 and page listings for all study components can be found in our Standard Protocol Items: Recommendations for Interventional Trials (SPIRIT) Checklist (see Additional file 1).

**Table 1:** Schedule of enrollment, interventions, and assessments

	Recruitment	Enrollment	Allocation	Intervention	Exit
Time point	−*t2*	−*t1*	0	*t1*	*t2*
Enrollment:					
Eligibility screen	X	X			
Informed consent		X			
Intake interview		X			
Allocation			X		
Interventions:					
* CHW* + *SS*				X	
* SS* + *M*				X	
Assessments:					
Cervical cancer screening		X			X
Sociodemographic and psychosocial characteristics		X			X
Cervical cancer knowledge, access to care, knowledge and follow-up of test results, acceptability of screening delivery		X			X

*CHW + SS* CHW self-sampling intervention, *SS + M* Self-sampling by mail

Our study occurs in three South Florida immigrant communities: Little Haiti, Hialeah, and South Dade. More than 100,000 Haitian immigrants are known to reside in Little Haiti, though the actual number may exceed 250,000 [25, 26]. The majority of Little Haiti residents were born in Haiti and speak a language other than English at home [7]. The median household income in the seven census tracts that comprise Little Haiti is US$21,646 and 45% of the population lives below the federal poverty limit [26]. Over two thirds of the adult population in Little Haiti does not have a high school diploma or GED [26]. Little Haiti is a federally designated Medically Underserved Area/Population (MUA/P) and Health Provider Shortage Area [27]. Access to care in this neighborhood may be complicated by limited health insurance coverage (only 40% of residents were covered prior to the Affordable Care Act), immigration status and fear of deportation, and limited proficiency in English and/or Spanish, the primary languages spoken in Miami, FL [28].

Our second community, Hialeah, is located in MiamiDade County and is part of the Miami metropolitan area. It is a historic ethnic enclave comprised largely of Cuban immigrants and Cuban Americans who represent 74% of the community’s 232,311 residents [29]. The median household income in Hialeah is US$33,942 and 26% of residents live below the federal poverty threshold [29]. Twenty-nine percent of adults in Hialeah do not have a high school diploma or equivalent [29]. A vast majority (91%) of adults speak Spanish at home [29]. English language proficiency is limited for many, which constitutes a barrier to seeking timely cancer and control prevention information, including information about routine screening [24–29].

The community of South Dade is located just south of Miami and has a total population of 208,491 [26]. Approximately 22% of residents identify as Black. The ethnic make-up of South Dade is primarily Hispanic (63%) of diverse national origins (e.g., 46% Cuban, 26% Central or South American, 10% Puerto Rican, 4% Dominican, and 13% Mexican) [26]. South Dade is a low-income area, with a median household income of approximately US$45,322; nearly a quarter (23%) of residents live below the federal poverty threshold [26]. South Dade is also a federally designated health provider shortage area, as well as a designated Medically Underserved Area/Population (MUA/P) [27].

**Fig. 1:**
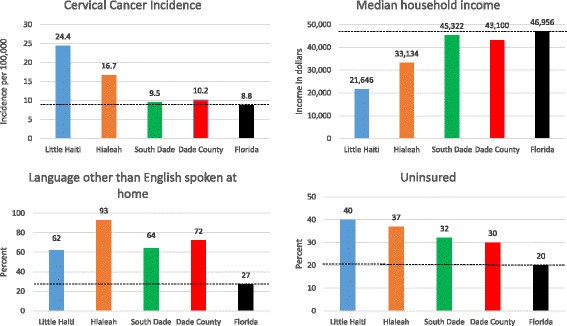
Cervical cancer incidence and sociodemographic characteristics in intervention communities

As indicated by the sociodemographic profiles of these three communities depicted in Fig. 1, poverty, low educational attainment, limited English language proficiency and limited access to care represent major challenges to public health initiatives that target cancer prevention. Additionally, sociopolitical histories that include discrimination and longstanding linguistic isolation further complicate access to routine cervical cancer screening by fostering communitywide distrust of medical authorities and research in general [7, 24]. Such distrust necessitates a responsive approach that works within the particular sociopolitical, linguistic, and cultural frameworks of these communities to promote cancer prevention [7, 24]. This approach thus demands guidance and collaboration with community partners from each site, as is consistent with community-based participatory research (CBPR).

#### Conceptual approach

Our approach is driven by Social Ecological Theory, which postulates the interaction between larger systemic factors (relationships, communities, culture, etc.) and individual factors influence health behavior [30]. Given this approach we recognize that the aforementioned barriers to cervical cancer screening among immigrant women exist not only on the individual level, but also on the larger systemic, cultural, and community levels; our approach aims to engage each of these levels. Thus, the development, design, and implementation of our study is guided by the principles of CBPR. CBPR engages communities at these multiple levels of influence through partnerships with key community stakeholders which inform the development of culturally-sensitive interventions and initiatives. Ultimately, through these partnerships CBPR allows for the dissemination of intervention not only at the individual but also at the community level. Prior to the SUCCESS trial, we developed campus-community partnerships with key community stakeholders in each of our target communities (Little Haiti, Hialeah, and South Dade) [7]. With these stakeholders, as well as with key community members, we formed Community Advisory Groups (CAGs) within each of the communities. These groups guided the goals, scope, and development of our prior SUCCESS trial as well as the current study, providing critical input at each step of the work from design to dissemination. Moreover, Health Choice Network, a federally-qualified health care center and one of our key community partners, is subcontracted on the study to hire and manage CHWs, as well as to provide any necessary follow-up care for study participants.

### Community health worker training

In response to the historical distrust of “outsiders” by community residents, key community leaders in Little Haiti, Hialeah, and South Dade suggested that study data should be collected by CHWs who are indigenous to the neighborhoods and knowledgeable about community norms and customs. Each community site employs and supervises one CHW. CHWs must be Hispanic or Haitian, have graduated from college, and speak English and Haitian Creole or English and Spanish fluently.

The principal investigator (PI) along with key community partners developed formalized in-person research training for the CHWs. The training was provided by the study manager. This training is based on the principles of participatory learning and adult education, and is organized into four modules. The first session covered the epidemiology of cervical cancer, and introduced the CHWs to study aims and their role in accomplishing such aims. The second session taught the CHWs about the principles of research ethics, and helped them to complete the University of Miami’s certification course (CITI) for conducting human subject research. The third and fourth sessions provided rigorous instruction on research methodology and collecting study data. As part of these sessions, the CHWs formally practiced and received feedback on their interviewing and teaching skills.

### Eligibility

#### Inclusion criteria

To be eligible women must be: (1) Haitian, Hispanic or African American, (2) aged 30–65 years, (3) report not having had a Pap smear in the last 3 years, and (4) live in the neighborhoods of Miami/Little Haiti, Hialeah or unincorporated Southern MiamiDade. Women under the age of 30 years have a high false-positive HPV rate due to transient HPV infections. Thus, the HPV test is not recommended for that age group [31, 32]. Our older age cutoff of 65 years is based on the Unites States Preventive Services Task Force (USPSTF) cervical cancer screening recommendations [33].

#### Exclusion criteria

Women are not included in the study if they: (1) report having had a hysterectomy, (2) have a history of cervical cancer, (3) plan to move out of the neighborhood during the 6 months after enrollment, and/or (4) are enrolled in any other cancer prevention/outreach-related study.

### Allocation of trial interventions

Once participants complete the enrollment interview, they will be randomized into one of two possible interventions. This randomization will be done by the study biostatistician, using REDCap’s randomization module. This module allows for the electronic randomization of study participants to study arm, with the option of randomizing within study sites to account for nesting. As our study design is hierarchical, with each subject nested within a site, the randomization is based at the site level (i.e., participants will be randomized within site). Once randomization is completed, the CHWs are notified by an automated email generated by REDCap. The CHWs then follow up with the women within a week, inform them of their assignment and, if in the control arm (CHW + SS), schedule the home visit. The CHE who conducts the enrollment and 6-month follow-up visit is blinded to participant group assignment. Both the CHWs and the participants are aware of study arm assignment.

### Recruitment

Recruitment occurs at various community venues, including laundromats, churches, health clinics, and flea markets throughout Little Haiti, Hialeah, and South Dade. The CHWs also rely heavily on their own social networks and those of the community leaders, active in SUCCESS CAGs, to identify potentially eligible participants. Our preliminary research in Little Haiti and Hialeah indicates that random recruitment strategies engender suspicion and compromise overall participation. As a result, we must employ a nonprobability, purposive quota-based sampling strategy to recruit participants. We will compare study samples with data abstracted from the US census to better understand whether our sample is, in fact, representative of our target communities. The CHW will approach potentially-eligible individuals at community venues, introduce themselves, and explain their role as a CHW. They will then inquire if a potential participant has recently been screened for cervical cancer, and if not, if they would be interested in participating in a study. The CHWs then screen interested potential participants for eligibility, and for those women who screen eligible, the CHW further describes the study. For eligible and interested women, the CHW tries up to ten times to reach a potential participant over a month to schedule the informed consent/interview with the CHE.

### The intervention

#### HPV self-sampling device

For this study, we plan to use a self-sampling device developed by Preventive Oncology International (POI) and the National Institutes of Health (NIH). The POI/NIH self-sampler is 20 cm in diameter, which is similar to a regular-sized tampon. This sampler is easy to use. A woman inserts the device into her vagina until she feels slight resistance, exposing the collection swab tip to the cervix. The participant then holds the paddle end of the collection swab and rotates it three times. She then withdraws the collection swab. Once the collection swab is fully removed from the vagina, the participant (or health worker) places the collection swab into liquid cell preservative. HPV testing can be effectively performed on this single specimen. Consistently this device has been found to be just as sensitive as a physician-performed test for detecting HPV and, in some instances, cervical dysplasia [20, 21].

#### Intervention group 1—CHW-delivered HPV self-sampling (CHW + SS)

In general, the intervention strategy involves two overlapping domains: (1) health education on cervical cancer screening and (2) instructing women about how to appropriately self-sample. They are also advised that if HPV obtained through self-sampling is positive, they will need follow-up medical care at our clinical sites. The CHWs stays with a woman (not in the same room) as she self-samples in order to answer any questions that may arise during the process. The samples can be stored at room temperature and are delivered once a week to a CLIA-approved laboratory for testing (APTIMA HPV Assay; GenProbe Inc.). Women who choose not to self-sample are provided with information about our community partners and other local resources where they can get a free and/or low-cost Pap smear.

#### Intervention group 2—mailed HPV self-sampling

Within 1 week after completing the interview, women randomized to the intervention arm receive a self-sampling kit via mail. This kit includes: (1) the self-sampler, (2) the vial for collecting and storing the specimen, (3) a preaddressed, stamped envelope for returning the vial to the CLIA-approved laboratory for HPV testing (see above), (4) a brief introductory letter which includes a picture of the CHE and the CHW and serves to reorient the participant to the focus of the study, (5) a brochure containing the same images that CHWs present to control group participants during home visits, that visually depict the steps for self-sampling (see Additional File 2), (6) NCI pamphlets about cervical cancer and the importance of early detection of disease in the participant’s language of preference (identified at enrollment), and (7) information provided by our community partner in each site about their organization, the medical services provided, and how to schedule a Pap smear. We are intentionally not having the CHW formally contact intervention participants before the 6-month mark to avoid unduly influencing their decision to self-sample. However, we are having the CHW make one phone call a week after the self-sampling kit has been mailed to confirm that the participant did, in fact, receive it.

### Community health workers

In response to the historical distrust of “outsiders” by community residents, key community leaders in Little Haiti, Hialeah, and South Dade suggested that study data should be collected by CHWs who are indigenous to the neighborhoods and knowledgeable about community norms and customs. Each community site employs and supervises one CHW. CHWs must be Hispanic or Haitian, have graduated from college, and speak English and Haitian Creole or English and Spanish fluently.

The PI along with key community partners developed formalized in-person research training for the CHWs. The training was provided by the study manager. This training is based on the principles of participatory learning and adult education, and is organized into four modules. The first session covered the epidemiology of cervical cancer, and introduced the CHWs to study aims and their role in accomplishing such aims. The second session taught the CHWs about the principles of research ethics, and helped them to complete the University of Miami’s certification course (CITI) for conducting human subject research. The third and fourth sessions provided rigorous instruction on research methodology and collecting study data. As part of these sessions, the CHWs formally practiced and received feedback on their interviewing and teaching skills.

### Trial procedures

#### Enrollment visit

The interview is conducted by our CHEs to avoid introducing bias into our study design. Prior to beginning the interview, the CHE first conducts another eligibility screen to verify that the potential participant still meets study eligibility criteria. The CHE will then explain for a second time the study to the potential participant and answer any questions she may have. If she is still agreeable to participating, the CHE obtains signed informed consent. After obtaining consent, the CHE proceeds with the interview (see Additional File 3). This interview is conducted using laptops with access to the REDCap web-based platform (see below). Backup paper copies are available in case the CHE experiences difficulty accessing the web.

Given widespread skepticism about research in our target communities, the need for brief measures has been one of the most important lessons we have learned from our experiences with participants. Thus, our interview is no more than 30 min in length. Our top priority is to collect data related to our primary outcome variable, cervical cancer screening. The second priority is to collect data on potential confounders that we may need to adjust for. Our third priority is to collect data on additional outcomes via our enrollment and exit questionnaires, including access to care, and cervical cancer knowledge, attitudes, and beliefs. Our interview draws from previously-validated scales, as well as our ongoing research with the target communities. Though originally conceived in English, we translated the questionnaire from English to Haitian Creole and Spanish, as well as tailored questionnaire items based on community feedback.

#### Follow-up interview at 6 months

At 6 months post enrollment, the CHE completes a follow-up interview with the participant (see Additional File 4). The follow-up interview contains the same content as the enrollment interview, with the addition of self-sampler acceptability items for those who have completed screening. The CHW schedules the follow-up interview with the CHE.

#### Participant timeline

We began enrollment in month 3 and will end enrollment by month 27 of our 36-month study (see Additional File 5). Thus, our recruitment goal is 10–12 women/month per site (30–36 women/month total). These assumptions are based on our prior experience with our SUCCESS trial where we recruited nearly 12 women/month at each site on average.

### Outcome measures

#### Primary outcome

Our primary outcome will be completion of HPV self-sampling within 6 months post enrollment. Using an intent-to-treat approach, we will assume that any women who are lost-to-follow-up did not complete cervical cancer screening.

#### Secondary outcomes

We also plan also to examine several secondary outcomes, including: (1) access to care (health insurance, having a usual source of care, and whether there has been a visit to a provider in 6 months), (2) cervical cancer knowledge, attitudes, and beliefs (measured using our initial and exit questionnaires developed in collaboration with community partners), (3) knowledge of test results and, for those with an abnormal screen, the proportion of women in each group who received appropriate follow-up within 30 days of the abnormal screen, and (4) acceptability of screening delivery.

### Sample size calculation

We based the proposed sample size on our work with our previous trial, SUCCESS. As previously mentioned, CHW with self-sampling (CHW + SS) was one of the study arms in that trial’s three-armed randomized study. Approximately 90% of women randomized to our CHW + SS arm complete screening as compared to 47% of women who receive CHW education and navigation to Pap smear screening. In the proposed study, we will introduce self-sampling through mail (SS + M) and compare screening uptake between this approach and CHW + SS.

With these considerations, the sample size was chosen to ensure sufficient power (80%) to detect clinically meaningful effects associated with a 20% point increase in proportion screened in the SS + M intervention group versus CHW + SS at an alpha of 0.05. The calculations performed indicate that the proposed total sample size of 600 women (i.e., 200 women at each site) will provide sufficient power for the main study hypotheses, under a variety of assumptions.

### Fidelity

We will assess intervention fidelity across each community through ongoing review of CHW participant encounter logs, which provide detailed process data regarding intervention delivery. Any identified inconsistencies in study implementation will be systematically documented, as well as addressed as part of ongoing team meetings. We will examine participant HPV knowledge post intervention as an additional measure of intervention fidelity, and also conduct a cost analysis. The observed differences in the cost of one arm relative to the other is necessary to inform future implementation and dissemination.

### Withdrawal of participants from study

Any participant may choose to withdraw from the study at any time. If a participant chooses to withdraw, their data will be destroyed, and their reason for withdrawal recorded. Participants may withdraw by contacting the CHE.

### Data management

All study data collected will be uploaded into the REDCap system. As previously stated, CHEs have access to this system to enter participant data in real time. REDCap is a web-based research management application that is designed specifically for investigators and their research teams. It supports processes for patient recruitment, scheduling, budgeting, invoicing, milestone management, data safety monitoring, adverse event reporting, system integration, data collection, and study execution. It is easy to use, reliable, fully HIPAA-compliant, and completely secure. CHEs responsible for data entry will be immediately notified, in REDCap, of missing fields and improbable values when entering data. Furthermore, quality and completeness of data entry will be systematically checked by a data manager at regular intervals for the duration of the study. Only the study statistician has access to the raw data files entered by CHWs. De-identified data files that have been cleaned and appropriately grouped and categorized will be made available to study personnel requesting such data.

### Statistical analysis

All of the statistical analyses will be carried out using SAS for Windows (Cary, NC, USA), Splus (Insightful, Inc.) for Windows, and SPSS for Windows. Initially, we will perform an exploratory data analysis and calculate descriptive statistics. Intent-to-treat analyses to examine uptake of cervical cancer screening will be conducted. All women who were randomized will be included in these analyses, and any participant who is lost-to-follow-up will be assumed not to have completed the cervical cancer screening. Associations between covariates and group assignment will be evaluated via logistic regression. Any covariates differing significantly between study groups will be controlled for when evaluating the association between group assignment and screening uptake via logistic regression.

#### Subgroup and secondary outcome analyses

We have several planned subgroup analyses to help us understand whether our intervention was more effective among certain population subgroups. We therefore propose to examine differences by site, ethnic group, education level, health insurance status, and acculturation level. To evaluate subgroup differences, we will model interactions between these variables and study group assignment, and present stratified analyses for those variables with significant interactions. For example, we will test whether the intervention effect differs across sites by modeling an interaction between site and group assignment, then stratify our primary analyses by study site if the interaction is significant in order to examine differences between study arms regarding cervical cancer screening uptake. We also plan to examine the change in several secondary outcomes, described previously, within the overall sample, as well as between study groups via repeated measures analysis of variance (ANOVA).

#### Cost analyses

As an additional secondary aim, we will estimate the cost of providing the intervention. From this, we can determine the additional cost of screening one woman in the SS + M as compared to the control arm (CHW + SS); this cost may be positive or negative. To do this, we will first estimate the total cost of providing the intervention arm to the 200 women recruited into that arm. We will then divide the total cost of that arm by the number of women screened to get a “cost per woman screened” for each of the arms. To calculate a total cost for each arm, we will collect resource utilization information on all aspects of the program. Using a comprehensive data collection form that we will develop in the first months of the program, we will collect information on all resource use that occurs during the intervention. Information that is collected includes: (1) personnel time: this includes personnel time that is devoted to training the CHEs and CHWs, assessing eligibility, managing contacts, and recruiting, conducting the intervention, and administrative time. We will not include the time that is spent on research activities, as this would not be applicable to a nonresearch community-based intervention, (2) space: we collect information on space used for the delivery of the intervention (where applicable) and administrative space, (3) supplies: we collect information on all supplies that are needed (e.g., the NCI pamphlets for both arms, mail costs), and (4) medical care: we have information on number of self-samplers analyzed and Pap smears completed.

#### Data monitoring

We perform primary outcome analyses every 6 months and report our findings to the NCI. Additionally, all study staff and the PI meet monthly to review study progress, as well as any adverse events and feedback from participants regarding study procedures. Moreover, we have developed quality control procedures to monitor all aspects of study implementation. Finally, study data is reviewed on a regular basis to ensure proper entry and storage procedures.

### Reporting and dissemination

#### Protocol approvals and amendments

The current study was approved by the University of Miami Institutional Review Board. There have been 12 minor amendments to update the study documents, as well as staff added to the protocol (version date: 21 June 2016).

#### Informed consent

As previously mentioned, two bilingual (English/Creole, English/Spanish) CHEs obtain informed consent in the participant’s preferred language (the Informed Consent Document is translated into Spanish and Haitian Creole). If a potential participant cannot read, the CHE reads the Informed Consent Document aloud to her, with a witness to ensure no bias in the consent process. CHEs answer all participant questions prior to obtaining their signatures.

#### Confidentiality

All participants are assigned a study ID number and study interview documents and datasets will be de-identified. Only study staff have access to the document that links study ID with participant names. All paper files are stored in locked file cabinets within secure office spaces. All electronic files are stored on secure servers on password-protected computers.

#### Financial interests

All of the study team members, as well as the study staff, have no financial interests to disclose.

#### Access to data

Only the data manager and study biostatistician have access to raw data files. The data manager may grant other study staff access to cleaned and de-identified datasets if needed, as well as generate tables and charts for ongoing data analysis and monitoring. Outside investigators may request access to de-identified datasets through the study PIs.

#### Post-trial care

For all women who remain unscreened at the 6-month follow-up, CHWs offer another opportunity to self-sample and/or linkage with a Pap smear provider, based on the preferences of the participant. All participants who screen positive for HPV are referred to low-cost follow-up care by the CHWs. CHWs will navigate participants who screen positive for HPV to one of our low-cost community health care providers, and follow-up with participants to ensure that appointments have been made and attended. These health care providers will determine the follow-up care (e.g., Pap smear, colposcopy) to be provided to participants.

#### Dissemination

In addition to disseminating our findings through journals, conferences, and other standard academic channels, our community partners will oversee the dissemination of research findings within our target communities. Within the community setting, we will disseminate findings through culturally appropriate communication channels, including local radio and by organizing a series of community forums.

## Discussion

Our project addresses the excess burden of cervical cancer experienced by Little Haiti, Hialeah, and South Dade, three medically underserved communities within the Miami metropolitan area [2–14]. Using a CBPR approach, we will determine the efficacy of CHW versus mail-delivered HPV self-sampling among 600 women from our target communities. Study findings will allow us to optimize this for cervical cancer screening in real-world settings.

The current study is not without methodological limitations, however. The use of one CHW per site may impede our ability to determine whether differences in study outcomes between our communities are attributable to community versus CHW-related factors. As such, we employ quality control measures to ensure study fidelity and optimize reliability among our CHWs. Additionally, we collect data on each interaction that CHWs have with participants (including phone calls), to better understand the potential impact of CHW-related factors on study outcomes.

Additionally, while HPV testing has been shown to demonstrate greater sensitivity and negative predictive value than the Pap smear, it is not yet standard-of-care practice for primary cervical cancer screening [21]. However, our prior work indicates that many women within our target communities experience significant barriers to Pap smear screening that may be circumvented by HPV self-sampling. While the USPSTF does not yet recommend HPV self-sampling as the “gold standard” method, it does recommend the use of this method among hard-to-reach populations [33]. In our previous SUCCESS trial, many women who were not amenable to Pap smear screening elected to self-sample, and through their engagement with our study, eventually were able to connect with the formalized health care system. As with our prior trial, all women in the current study are given information and referrals to community health centers where they may receive low-cost Pap smear screening.

## Trial status

As of 25 August 2016, we have enrolled 571 of our target 600 participants in the study.

## Additional files

Additional file 1: SPIRIT Checklist (.docx): page listings for all SPIRIT study components. (DOC 122 kb)
Additional file 2: Self-sampling instructions. (PDF 100 kb)
Additional file 3: Enrollment questionnaire. (DOCX 480 kb)
Additional file 4: Exit questionnaire. (DOCX 232 kb)
Additional file 5: Trial flow diagram. (DOCX 153 kb)

## Abbreviations

CAG: Community Advisory Group
CBPR: Community-based participatory research
CHE: Community health educator
CHW + SS: Community health worker + self-sampling intervention, group 1
HCN: Health Choice Network of Florida
HPV: Human papilloma virus
NCI: National Cancer Institute
SS + M: Self-sampling + mail intervention, group 2
SUCCESS: South Florida Center for the Reduction of Cancer Health Disparities
US: United States
USPSTF: Unites States Preventive Services Task Force
