# Supplementary material for: Mailed HPV self-sampling for cervical cancer screening among underserved minority women: study protocol for a randomized controlled trial
Source: Trials. 2017 Jan 13;18:19. doi: 10.1186/s13063-016-1721-6 (PMC5237204; doi:10.1186/s13063-016-1721-6)
Supplement: Additional file 2: — Self-sampling instructions. (PDF 100 kb) [file 13063_2016_1721_MOESM2_ESM.pdf]

Self-Sampler Instructions

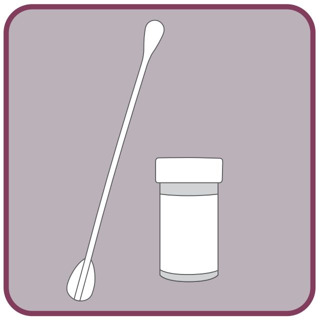

1 Inside the envelope you will find a swab and a jar.

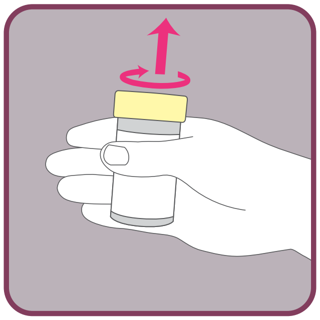

2 Open the jar.

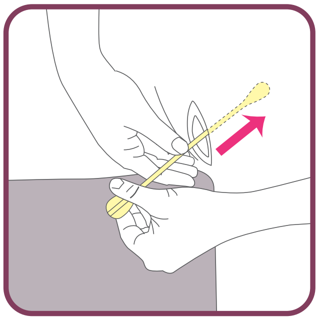

3 Put the swab into your vagina with the cotton end pointed toward you.

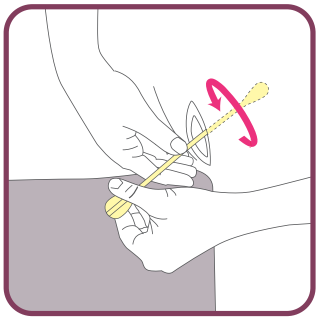

4 Push the swab until you feel it can't go any further. Turn the swab 5 times.

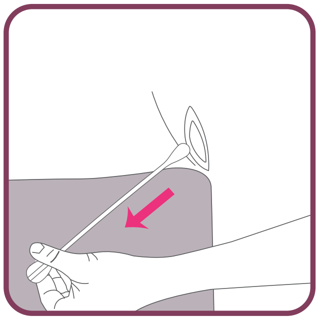

5 Remove the swab from your vagina.

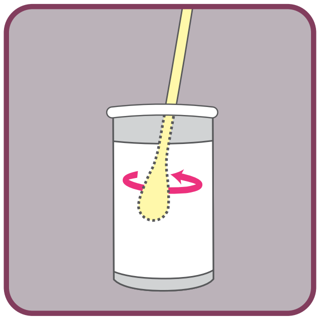

6 Put the cotton end of the swab into the jar and stir it 5 times in the liquid.

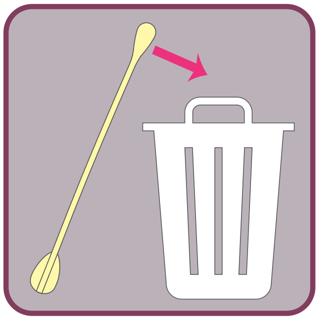

7 Take the swab out of the jar and throw the swab in the trash.

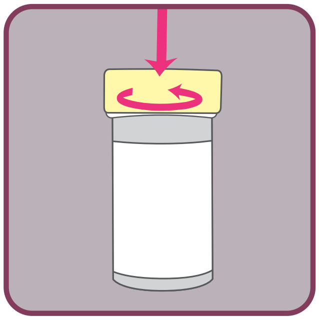

8 Put the lid on the jar. Make sure the lid is tightly closed.

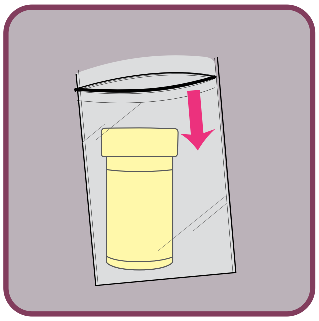

9 Put the jar back in the plastic bag.

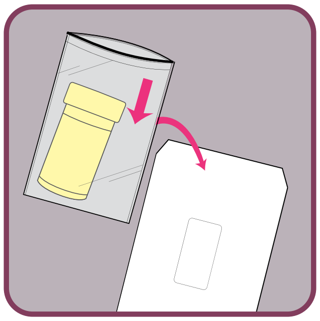

10 Put the bag in the pre-paid envelope.

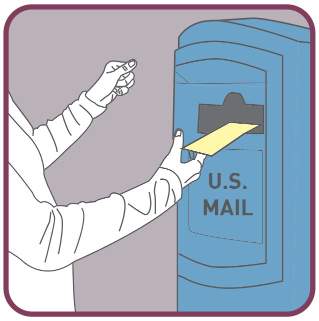

11 Seal the pre-paid envelope and mail it back to us.

We will call you with your results!
