# Supplementary material for: Mailed HPV self-sampling for cervical cancer screening among underserved minority women: study protocol for a randomized controlled trial
Source: Trials. 2017 Jan 13;18:19. doi: 10.1186/s13063-016-1721-6 (PMC5237204; doi:10.1186/s13063-016-1721-6)
Supplement: Additional file 3: — Enrollment questionnaire. (DOCX 480 kb) [file 13063_2016_1721_MOESM3_ESM.docx]

**
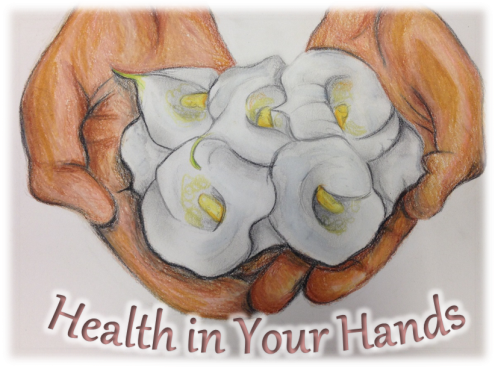
Intake Survey: (done by CHEs in person)**

**Date: ________**

**Location: Home _________________________**

**Other (specify): __________________________**

**Start Time: _____________**

***THANK YOU FOR AGREEING TO BE A PART OF THIS STUDY. IN THIS INTERVIEW, WE ARE INTERESTED IN HEARING ABOUT YOUR LIFE EXPERIENCES AS WELL AS YOUR OPINIONS REGARDING SEVERAL HEALTH TOPICS IMPORTANT TO WOMEN AND THEIR FAMILIES. THE INFORMATION YOU SHARE WITH US WILL BE KEPT COMPLETELY CONFIDENTIAL, AND YOU CAN REFUSE TO ANSWER ANY QUESTION AT ANY POINT DURING THE INTERVIEW. WE WILL USE THE INFORMATION YOU SHARE WITH US TO PLAN FUTURE EFFORTS TO PROMOTE HEALTH AND WELL-BEING AMONG WOMEN IN YOUR COMMUNITY.***

***Language preference:***

1. **In what language would you prefer this survey?**

- English
- Spanish **(STOP HERE. Use version B for Spanish)**
- Creole **(STOP HERE. Use version C for Creole)**

***Ethnicity and race questions:***

1. **Are you Hispanic/Latina?**

- Yes
- No

1. **How would you describe your race?**

(If does not volunteer, ask respondent: **Well, do you consider yourself ____?** Offer choices)

(If still does not respond, say:  **This question is also often asked in the Census form, do you recall what you answered in the Census.)**

NOTE: Respondent may provide more than one response, check all that apply

- White
- Black
- Mixed (interracial, mulatto, creole, etc)
- Asian
- Native Hawaiian or Pacific Islander
- American Indian or Alaska Native
- Other (Specify ____)
- Unknown
- Refuse to answer

1. **Were you born the United States or another country?**

Yes, United States ……………………………………………..1

No, other Country ……………………....................................2

Country_____________________

Unknown ………………………………………………………..3

Refuse to answer……………………………………………….4

1. **At what age did you immigrate to the United States?**

Age < 18 …………………………………………………..……1

Age > 18…………………………………………………………2

Unknown ………………………………………………………..3

Refuse……………………………………………………………4

1. When (what year) did you immigrate to the United States?__________

**Now, I am going to ask you some questions about the Pap smear test.**

1. **You have indicated that you have not had a Pap smear in the last three years. But, have you ever had a Pap smear or Pap test?**

YES 1 **(SKIP TO 10 )**

NO 2

DON’T KNOW 3

1. **What is the main reason you have not have had a PAP smear:**

***If respondent hesitates, read all choices. single answer***

Do not know what that is ………………………………………………………….1

You did not want to have one……………………………………………………..2

You did not know you needed one ……………………………………………….3

Too busy to have one done ……………………………………………………….4

You couldn't afford it or did not have health insurance……………………….…5

You couldn't get an appointment ………………………………………….…...…6

The (clinic/doctor's) office are not open when you could get there……..……...7

You didn't have transportation…………………………………………….……….8

Other (SPECIFY)…………………………………………………………………...9

**(SKIP TO 14 )**

1. **Approximately when was your last Pap smear?** |_____||_____| MONTH/YEAR
2. **What was the main reason you had this Pap smear?** **(DO NOT READ RESPONSES)**

ROUTINE ANNUAL PAP SMEAR OR PART OF

ROUTINE PHYSICAL EXAM 1

LAST PAP SMEAR WAS NOT NORMAL 2

A SPECIFIC PROBLEM 3

SOMETHING SHE HEARD / SAW / READ 4

SHE HAD NEVER HAD ONE AND THOUGHT SHE SHOULD 5

PREGNANCY / FOLLOW-UP TO BIRTH 6

OTHER (SPECIFY)…. ………. 7

1. **Have you ever been told by a healthcare provider that your Pap smear was abnormal?**

YES 1

NO 2 (**SKIP TO 14)**

DON’T KNOW 3

1. **What did you do about it?** **(DO NOT READ RESPONSES)**

NOTHING 1

REPEAT PAP SMEAR, THEN NORMAL 2

TREATED FOR CERVICAL DYSPLASIA USING FREEZING 3

UNDERWENT COLPOSCOPY & BIOPSY, THEN NORMAL 4

OTHER (SPECIFY)_________________________________................... 5

DON’T KNOW 6

1. **How often do you think a woman your age should have a Pap smear?**

MORE THAN TWICE A YEAR 1

TWICE A YEAR / EVERY SIX MONTHS 2

ONCE A YEAR 3

EVERY TWO YEARS 4

EVERY THREE YEARS 5

MORE THAN EVERY THREE YEARS 6

NEVER 7

DON’T KNOW…….……….. ….. 8

1. **Do you think that when you stop having children you no longer need to have Pap smears?**

YES 1

NO 2

DON’T KNOW 3

**Now I will be asking you some questions about your knowledge of cervical cancer.**

1. **Do you think that cervical pre-cancers and early cancers show symptoms or signs?**

YES 1

NO 2

DON’T KNOW 3

1. **Do you think that multiple abortions can cause cervical cancer?**

YES 1

NO 2

DON’T KNOW 3

1. **Do you think that being hit in your lower abdomen can cause cervical cancer?**

YES 1

NO 2

DON’T KNOW 3

1. **Do you think that having a high number of sexual partners increases your risk for cervical cancer?**

YES 1

NO 2

DON’T KNOW 3

1. **Do you think that women who smoke are more likely to develop cervical cancer than non-smokers?**

YES 1

NO 2

DON’T’ KNOW 3

**Now I will be asking you some questions about the Human Papillomavirus, also known as HPV. HPV is NOT HIV.**

1. **Have you ever heard of HPV? HPV stands for Human Papillomavirus.**

YES 1

NO 2 **(SKIP TO 24)**

DON’T KNOW 3

1. **Do you think that HPV can cause cervical cancer?**

YES 1

NO 2

DON’T KNOW 3

1. **Do you think that you can get HPV through sexual contact?**

YES 1

NO 2

DON’T KNOW 3

**Now I will ask you some questions about your health insurance.**

1. **Do you have Health Insurance? (Jackson health card is not having insurance)**

YES 1

NO 2

DON’T KNOW 3

Refuse to answer ………………………………………………………………. 4

Not Applicable ………………………………………………………………….. 5

1. **Do you have Private Health Insurance (such as insurance you get from where you work)?**

YES 1

NO 2

DON’T KNOW 3

Refuse to answer……………………………………….………………………. 4

Not Applicable (i.e, do not work) ……………………….…………………….. 5

1. **Do you have Medicaid?**

YES 1

NO 2

DON’T KNOW 3

Refuse to answer ………..………………………………………………………. 4

Not Applicable ………………………………………………………………….. 5

1. **Do you have Medicare?**

YES 1

NO 2

DON’T KNOW 3

Refuse to answer ………………………………………………………………. 4

Not Applicable …………………………………………………………………. 5

1. **Do you have other sources to help pay for your medical care?**

YES 1

NO 2

DON’T KNOW 3

Refuse to answer ………………………………………………………………. 4

Not Applicable ………………………………………………………………….. 5

1. **Specify other source :** ____________________

**Now I will ask you questions about where you get health care.**

1. **Is there a place that you USUALLY go to when you are sick?**

YES 1

NO 2 **(SKIP TO 32)**

DON’T KNOW 3 **(SKIP TO 32)**

Refuse to answer ………………………………………………………………. 4 **(SKIP TO 32)**

Not Applicable ………………………………………………………………….. 5 **(SKIP TO 32)**

1. **What kind of place is it - a clinic, doctor's office, emergency room, or some other place?**

– *IF more than one place, ask* **“What kind of place do you go to most often?*”***

Clinic or health center / Hospital outpatient (not emergency room) …1

Doctor's office ……………………………………………………...……..2

Hospital emergency room …………………………………………...…..3

Doesn't go to one place most often ………………………………...…..4

Refused ………………………………………………………………...….5

Don't know………………………………………………………………….6

Some other place _____________.......................................................7

1. **Is there a place that you USUALLY go to when you need routine or preventive care, such as a physical examination or check-up?**

YES 1

NO 2 **(SKIP to 34)**

DON’T KNOW 3 **(SKIP to 34)**

Refuse to answer ………………………………………………………………. 4 **(SKIP to 34)**

Not Applicable ………………………………………………………………….. 5 **(SKIP to 34)**

1. **What kind of place is it - a clinic, doctor's office, or some other place?**

Clinic or health center / Hospital outpatient (not emergency room) ….1

Doctor's office ……………………………………………………………..2

Hospital emergency room ………………………………………………..3

Refused …………………………………………………………………….4

Don't know……………………………………………………………….…5

Some other place _____________.......................................................6

1. **There are many reasons people delay getting medical care. In the PAST 12 MONTHS. Have you delayed getting care?**

Yes _____

No _____ **(SKIP to 36)**

1. **If so, for what reasons:**

You couldn't afford it…………………………………………………………..1

You couldn't get through on the telephone………………………………....2

You couldn't get an appointment soon enough…………………………….3

Once you get there, you have to wait too long to see the doctor…………4

The (clinic/doctor's) office wasn't open when you could get there………..5

You didn't have transportation to get there…………………………………6

1. **DURING THE PAST 12 MONTHS, HOW MANY TIMES have you gone to a HOSPITAL EMERGENCY ROOM about your own health (This includes emergency room visits that resulted in a hospital admission.)?**

None……………………………………………………………………………. 1

One or more times..................................................................................... 2

Refuse to answer …………………………………………………………….. 3

Don't know……………………………………………………………………… 4

1. **DURING THE PAST 12 MONTHS, HOW MANY TIMES have you seen a doctor or other health care professional about your own health?** **DO NOT INCLUDE TIMES YOU WERE HOSPITALIZED, VISITS TO EMERGENCY ROOMS, DENTAL VISITS, OR TELEPHONE CALLS.**

None…………………………………………………………………………….1

One or more times ………...........................................................................2

Number of times:_______________________________

Refuse to answer ……………………………………………………………...3

Don't know………………………………………………………………………4

**We would like to know how competent you feel in your ability to self-sample as part of this study. The following questions address how you feel about self-sampling.**

1. **Do you believe you are able to self-sample correctly?**

Not at all…………………………………………………………………………………………………1

Somewhat ………………………………………………………………………………………………4

Definitely…………………………………………………………………………………………………7

1. **Do you know what the self-sampler is testing for?**

Not at all…………………………………………………………………………………………………1

Somewhat ………………………………………………………………………………………………4

Definitely…………………………………………………………………………………………………7

1. **Do you understand why self-sampling is important to prevent cervical cancer?**

Not at all………………………………………………………………………………………………….1

Somewhat ………………………………………………………………………………………………4

Definitely…………………………………………………………………………………………………7

1. **Do you understand the connection between HPV and cervical cancer?**

Not at all………………………………………………………………………………………………….1

Somewhat ………………………………………………………………………………………………4

Definitely…………………………………………………………………………………………………7

1. **Do you know what to do if you receive positive results?**

Not at all………………………………………………………………………………………………….1

Somewhat ………………………………………………………………………………………………4

Definitely…………………………………………………………………………………………………7

**The next questions ask about your previous experiences with healthcare practitioners (doctors, nurses, etc). Instead of thinking about just one practitioner, please answer about your previous experiences overall.**

1. **Do you feel that your health-care practitioners have provided you with choices and options about staying healthy?**

Not at all………………………………………………………………………………………………….1

Somewhat ………………………………………………………………………………………………4

Definitely…………………………………………………………………………………………………7

1. **Do you feel your health-care providers understand how you see things with respect to your health?**

Not at all………………………………………………………………………………………………….1

Somewhat ………………………………………………………………………………………………4

Definitely…………………………………………………………………………………………………7

1. **Do you feel your health-care providers convey confidence in your ability to make changes regarding your health?**

Not at all………………………………………………………………………………………………….1

Somewhat ………………………………………………………………………………………………4

Definitely…………………………………………………………………………………………………7

1. **Do you feel your health care practitioners listen to how you would like to do things regarding your health?**

Not at all………………………………………………………………………………………………….1

Somewhat ………………………………………………………………………………………………4

Definitely…………………………………………………………………………………………………7

1. **Do you feel your health-care practitioners encourage you to ask questions about your health?**

Not at all………………………………………………………………………………………………….1

Somewhat ………………………………………………………………………………………………4

Definitely…………………………………………………………………………………………………7

1. **Do you feel your health-care practitioners try to understand how you see your health before suggesting any changes?**

Not at all………………………………………………………………………………………………….1

Somewhat ………………………………………………………………………………………………4

Definitely…………………………………………………………………………………………………7

1. **Do you feel your health-care practitioners try to understand how your cultural values relate to your health?**

Not at all………………………………………………………………………………………………….1

Somewhat ………………………………………………………………………………………………4

Definitely…………………………………………………………………………………………………7

1. **Do you feel your health-care practitioners have been respectful of your beliefs and traditions as they relate to your health?**

Not at all………………………………………………………………………………………………….1

Somewhat ………………………………………………………………………………………………4

Definitely…………………………………………………………………………………………………7

**Now I will ask you questions about your language use and cultural preferences. If the participant is not Latina, please skip to question 75.**

1. **In general, what languages do you read in and speak?**

Only Spanish/Only Creole……………………………………………………………………………………1

Spanish/Creole better than English………………………………………………………………………2

Both Equally…………………………………………………………………………………………….. .3

English better than Spanish/Creole…………………………………………………………………………….4

Only English………………………………………………………………………………………………5

1. **What was the language you used as a child?**

Only Spanish/Creole……………………………………………………………………………………………..1

Spanish/Creole better than English …………………………………………………………………………....2

Both Equally…………………………………………………………………………………………….. .3

English better than Spanish/Creole…………………………………………………………………………….4

Only English………………………………………………………………………………………………5

1. **What language(s) do you usually use at home?**

Only Spanish/Creole……………………………………………………………………………………………..1

Spanish/Creole better than English …………………………………………………………………………....2

Both Equally…………………………………………………………………………………………….. .3

English better than Spanish/Creole…………………………………………………………………………….4

Only English………………………………………………………………………………………………5

1. **In which language(s) do you usually think?**

Only Spanish/Creole……………………………………………………………………………………………..1

Spanish/Creole better than English …………………………………………………………………………....2

Both Equally…………………………………………………………………………………………….. .3

English better than Spanish/Creole…………………………………………………………………………….4

Only English………………………………………………………………………………………………5

1. **What language(s) do you usually speak with your friends?**

Only Spanish/Creole……………………………………………………………………………………………..1

Spanish/Creole better than English …………………………………………………………………………....2

Both Equally…………………………………………………………………………………………….. .3

English better than Spanish/Creole…………………………………………………………………………….4

Only English………………………………………………………………………………………………5

1. **In what language(s) are the T.V. programs you usually watch?**

Only Spanish/Creole……………………………………………………………………………………………..1

Spanish/Creole better than English …………………………………………………………………………....2

Both Equally…………………………………………………………………………………………….. .3

English better than Spanish/Creole…………………………………………………………………………….4

Only English………………………………………………………………………………………………5

1. **In what language(s) are the radio programs you usually listen to?**

Only Spanish/Creole……………………………………………………………………………………………..1

Spanish/Creole better than English …………………………………………………………………………....2

Both Equally…………………………………………………………………………………………….. .3

English better than Spanish/Creole…………………………………………………………………………….4

Only English………………………………………………………………………………………………5

1. **In general, in what language(s) are the movie, T.V., and radio programs you prefer to watch or listen to?**

Only Spanish/Creole……………………………………………………………………………………………..1

Spanish/Creole better than English …………………………………………………………………………....2

Both Equally…………………………………………………………………………………………….. .3

English better than Spanish/Creole…………………………………………………………………………….4

Only English………………………………………………………………………………………………5

1. **Your close friends are:**

All Latinos/Hispanics or Haitian…………………………………………………………………………………….1

More Latinos/Haitian than Americans ………………………………………………………………………….2

About Half and Half……………………………………………………………………………………...3

More Americans than Latinos or Haitians…………………………………………………………………………..4

All Americans……………………………………………………………………………………………..5

1. **You prefer going to social gathering/parties at which the people are:**

All Latinos/Hispanics or Haitians…………………………………………………………………………………….1

More Latinos/Haitians than Americans ………………………………………………………………………….2

About Half and Half……………………………………………………………………………………...3

More Americans than Latinos or Haitians…………………………………………………………………………..4

All Americans……………………………………………………………………………………………..5

1. **The persons you visit or who visit you are:**

All Latinos/Hispanics or Haitians…………………………………………………………………………………….1

More Latinos/Haitians than Americans ………………………………………………………………………….2

About Half and Half……………………………………………………………………………………...3

More Americans than Latinos/Haitians…………………………………………………………………………..4

All Americans……………………………………………………………………………………………..5

1. **If you could choose your children’s friends, you would want them to be:**

All Latinos/Hispanics or Haitians…………………………………………………………………………………….1

More Latinos/Haitians than Americans ………………………………………………………………………….2

About Half and Half……………………………………………………………………………………...3

More Americans than Latinos/Haitians…………………………………………………………………………..4

All Americans……………………………………………………………………………………………..5

**The next set of questions is about your educational experiences.**

1. **How many years of school were you able to complete?**

No schooling…………………………………………………………………….1

Completed less than 4 years………………………………………………….2

Completed less than 8 years………………………………………………….3

Completed less than 12 years (some high school)…………………………4

Completed 12 years only (high school only)…………………………………5

More than 12 years of school………………………………………………….6

Unknown…………………………………………………………………………7

Refuse to answer………………………………………………………… ……8

Not Applicable……………………………………………………………………9

1. Record Number of Years: ________
2. **What was the grade you last completed?**  _________________

**Now I have some questions that ask about smoking.**

1. **Do you currently smoke cigarettes?**

YES 1

NO 2 **(SKIP TO 81)**

DON’T KNOW 3 **(SKIP TO 81)**

Refuse to answer ……………………………………………………...…………. 4 **(SKIP TO 81)**

1. **IF yes, how many cigarettes per day?**

< 10 per day…(half a pack)…………………………………….1

10-20 per day (about a pack)………………………………… 2

More than a pack ……………………………………………….3

Don’t Know……………………………………………………....4

Refuse to answer …………………………………………….. 5

1. **Have you smoked in the past?**

YES 1

NO 2

DON’T KNOW 3

Refuse to answer ………………………………………….……………………. 4

**Now I have some questions about your background. You do not have to respond to the following questions if you do not feel comfortable in doing so.**

1. **What is your marital status:** (OFFER CHOICES)

Single/Never married …………………………………………1

Living with significant other …………………………………..2

Married ………………………………………………………….3

Separated ……………………………………………………...4

Divorced ………………………………………………………..5

Widowed ……………………………………………………….6

Unknown ……………………………………………………….7

Refuse to answer ………………………………………………8

Not Applicable ………………………………………………….9

1. **What is your present employment status?**

Employed/self-employed………………………………………1

Retiree …………………………………………………………..2

Homemaker……………………………………………………..3

On disability……………………………………………………..4

Unemployed/not working………………………………………5

Unknown…………………………………………………………6

Refuse to answer ..……………………………………………..7

Not Applicable………………………………………………….. 8

**The following questions have to do with family finances. We know from other research that financial strain is common and very important to consider in understanding people's health. The following questions will be used to help give us a picture of the various financial situations experienced by persons participating in this study. Any information you provide is strictly confidential and will be used for research purposes only.**

If hesitant, ask and record yearly income: _____________

1. **I will read a list of income groups. Please tell me which group best represents your total combined family income for the past 12 months.**

Less than $3,000…………………………………………….1

$3,001 - $5,000 ……………………………………………...2

$5,001 - $10,000……………………………………………..3

$10,001 - $15,000……………………………………………4

$15,001 - $20,000 …………………………………………..5

$20,001 - $30,000 …………………………………………..6

$30,001 - $40,000……………………………………………7

$40,001 - $50,000……………………………………………8

$50,001 - $60,000 …………………………………………...9

Greater than $60,000 ………………………………………10

Unknown ……………………………………………………11

Refuse to answer ………………………...…………………12

Not Applicable……………………………………………….13

1. **This question is about the house or apartment where you live. Do you:**

Rent an apartment/ a home………………………………1

Rent Room…………………………………………………2

Pay a mortgage on a house you own…………………...3

Own house with no mortgage (free and clear) ……......4

Have other living arrangements ………………………….5

Unknown …………………………………………………..6

Refuse to answer ………………………..……………….7

Not Applicable …………………………………………….8

1. **How many people are living or staying at where you live? _________**

INCLUDE everyone who is living or staying here for more than 2 months.

INCLUDE yourself if you are living here for more than 2 months.

INCLUDE anyone else staying here who does not have another place to stay, even if they are here for 2 months or less.

DO NOT INCLUDE anyone who is living somewhere else for more than 2 months, such as a college student living away or someone in the Armed Forces on deployment.

**Again, you do not have to respond to the following questions if you do not feel comfortable in doing so.**

1. **Are you a United States citizen?**

YES ……………………………………………… 1  **(End of Survey: *Skip to exit script)***

NO ……………………………………….……. 2

DON’T KNOW…………………………………. 3

Refuse to answer ………………..…………… 4

1. **Do you have permanent resident status (also known as having a “green card”)?**

YES 1

NO 2

DON’T KNOW 3

Refuse to answer ……………………………………………………….……… 4

***EXIT: THOSE ARE ALL THE QUESTIONS I HAVE FOR YOU. (GIVE INCENTIVE TO PARTICIPANT) THANK YOU AGAIN FOR YOUR TIME AND PARTICIPATION IN THIS PROJECT. HERE IS MY CONTACT INFORMATION IF YOU HAVE QUESTIONS AT ANY TIME.***

**End Time: __________________**

**Survey completed:**

- Yes
- No

**Reasons why not: *_____________________________________________***

**Was cervical cancer and local provider information given?**

- Yes
- No
