# Supplementary material for: Mailed HPV self-sampling for cervical cancer screening among underserved minority women: study protocol for a randomized controlled trial
Source: Trials. 2017 Jan 13;18:19. doi: 10.1186/s13063-016-1721-6 (PMC5237204; doi:10.1186/s13063-016-1721-6)
Supplement: Additional file 4: — Exit questionnaire. (DOCX 232 kb) [file 13063_2016_1721_MOESM4_ESM.docx]

**
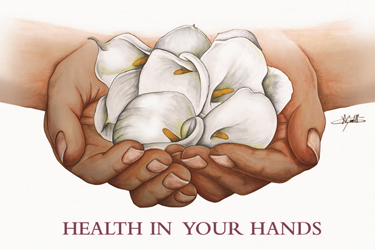
Exit Survey: (done by CHEs in person)**

**Date: ________**

**Location: Home _________________________**

**Other (specify) _________________________**

**Start Time: _____________**

***Language preference:***

1. **In what language would you prefer this survey?**

 English

 Spanish **(STOP HERE. Use version B for Spanish)**

 Creole **(STOP HERE. Use version C for Creole)**

1. **At the start of this study, you indicated that you have not had a Pap smear. Since the time you entered this study, have you had testing for the HPV virus through a vaginal swab/applicator? (if needed show picture)**

YES 1 **(SKIP TO 4 )**

NO 2

DON’T KNOW 3

1. **What is the main reason you have not had a HPV self-sampler test:**

***If respondent hesitates, read all choices. single answer***

Do not know what that is ……………………………………………………………..………1

You did not want to have one…………………………………………………………………2

You did not know you needed one …………………………………………………………..3

Too busy to have one done …………………………………………………………………..4

You were uncomfortable using the self-sampling kit…………………………………..……5

You were unsure about what the self-sampler was testing for …………………….….......6

You were unsure of what to do if the results were positive ……………………..………..7

Other: ___________________________________________ …………………………….8

1. **Was your HPV self-sampler test result normal?**

YES 1 (**SKIP TO 6)**

NO 2

DON’T KNOW 3

1. **What did you do about it?** **(DO NOT READ RESPONSES)**

NOTHING……………………………………………………………………………….1

REPEAT PAP SMEAR ……………………………………..…………………………2

TREATED FOR CERVICAL DYSPLASIA USING FREEZING,

CONE BIOPSY, OR LEEP………………………………………………………….....3

UNDERWENT COLPOSCOPY & BIOPSY ………………………………………….4

OTHER:________________________________________ ………………………5

DON’T KNOW …………………………………………………………………………..6

**The next set of questions is similar to the questions we asked you six months ago and ask about your knowledge of Pap smears.**

1. **How often do you think a woman your age should have a Pap smear?**

MORE THAN TWICE A YEAR 1

TWICE A YEAR / EVERY SIX MONTHS 2

ONCE A YEAR 3

EVERY TWO YEARS 4

EVERY THREE YEARS 5

MORE THAN EVERY THREE YEARS 6

NEVER 7

DON’T KNOW…….……….. ………………………………….. 8

1. **Do you think that when you stop having children you no longer need to have Pap smears?**

YES 1

NO 2

DON’T KNOW 3

**Now I will be asking you some questions about your knowledge of cervical cancer.**

1. **Do you think that cervical pre-cancers and early cancers show symptoms or signs?**

YES 1

NO 2

DON’T KNOW 3

1. **Do you think that multiple abortions can cause cervical cancer?**

YES 1

NO 2

DON’T KNOW 3

1. **Do you think that being hit in your lower abdomen can cause cervical cancer?**

YES 1

NO 2

DON’T KNOW 3

1. **Do you think that having a high number of sexual partners increases your risk for cervical cancer?**

YES 1

NO 2

DON’T KNOW 3

1. **Do you think that women who smoke are more likely to develop cervical cancer than non-smokers?**

YES 1

NO 2

DON’T’ KNOW 3

**Now I will be asking you some questions about the Human Papilloma virus, also known as HPV. HPV is NOT HIV.**

1. **Have you ever heard of HPV? HPV stands for Human Papilloma virus.**

YES 1

NO 2 **(SKIP TO 18)**

DON’T KNOW 3

1. **Do you think that HPV can cause cervical cancer?**

YES 1

NO 2

DON’T KNOW 3

1. **Do you think that you can get HPV through sexual contact?**

YES 1

NO 2

DON’T KNOW 3

**Now I will ask you some questions about your health insurance.**

1. **Do you have Health Insurance? (Jackson health card is not having insurance)**

YES 1

NO 2

DON’T KNOW 3

Refusal …………………………………………………………. 4

Not Applicable ………………………………………………….. 5

1. **Do you have Private Health Insurance (such as insurance you get from where you work)?**

YES 1

NO 2

DON’T KNOW 3

Refusal …………………………………………………………. 4

Not Applicable (i.e, do not work) …………………………….. 5

1. **Do you have Medicaid?**

YES 1

NO 2

DON’T KNOW 3

Refusal …………………………………………………………. 4

Not Applicable ………………………………………………….. 5

1. **Do you have Medicare?**

YES 1

NO 2

DON’T KNOW 3

Refusal …………………………………………………………. 4

Not Applicable ………………………………………………….. 5

1. **Do you have other sources to help pay for your medical care?**

YES 1

NO 2

DON’T KNOW 3

Refusal …………………………………………………………. 4

Not Applicable ………………………………………………….. 5

1. **Specify other source**

_________________________________________________________

**Now I will ask you questions about where you get health care.**

1. **Is there a place that you USUALLY go to when you are sick?**

YES 1

NO 2 **(SKIP TO 28)**

DON’T KNOW 3 **(SKIP TO 28)**

Refused …………………………………………………………. 4 **(SKIP TO 28)**

Not Applicable ………………………………………………….. 5 **(SKIP TO 28)**

1. **What kind of place is it - a clinic, doctor's office, emergency room, or some other place?**

– *IF more than one place, ask* **“What kind of place do you go to most often?*”***

Clinic or health center / Hospital outpatient (not emergency room) 1

Doctor's office ………………………………………………………..2

Hospital emergency room …………………………………………..3

Doesn't go to one place most often ………………………………..4

Refused ……………………………………………………………….5

Don't know…………………………………………………………….6

Some other place _____________................................................7

1. **Is there a place that you USUALLY go to when you need routine or preventive care, such as a physical examination or check-up?**

YES 1

NO 2 **(SKIP to 26)**

DON’T KNOW 3 **(SKIP to 26)**

Refusal …………………………………………………………. 4 **(SKIP to 26)**

Not Applicable ………………………………………………….. 5 **(SKIP to 26)**

1. **What kind of place is it - a clinic, doctor's office, or some other place?**

Clinic or health center / Hospital outpatient (not emergency room) 1

Doctor's office ………………………………………………………..2

Hospital emergency room …………………………………………..3

Refused ……………………………………………………………….4

Don't know…………………………………………………………….5

Some other place _____________................................................6

1. **There are many reasons people delay getting medical care. In the PAST 6 MONTHS. Have you delayed getting care?**

Yes _____

No _____ (**SKIP to 28)**

1. **If so, for what reasons:**

You couldn't afford it……………………………………………………………1

You couldn't get through on the telephone……………………………….....2

You couldn't get an appointment soon enough…………………………….3

Once you get there, you have to wait too long to see the doctor…………4

The (clinic/doctor's) office wasn't open when you could get there………..5

You didn't have transportation to get there………………………………….6

1. **DURING THE PAST 6 MONTHS, HOW MANY TIMES have you gone to a HOSPITAL EMERGENCY ROOM about your own health (This includes emergency room visits that resulted in a hospital admission.)?**

None…………………………………………………………………………….1

One or more times.....................................................................................2

Number of times: ________

Refused ………………………………………………………………………...3

Don't know………………………………………………………………………4

1. **DURING THE PAST 6 MONTHS, HOW MANY TIMES have you seen a doctor or other health care professional about your own health?** **DO NOT INCLUDE TIMES YOU WERE HOSPITALIZED, VISITS TO EMERGENCY ROOMS, DENTAL VISITS, OR TELEPHONE CALLS.**

None…………………………………………………………………………….1

One or more times .............................................................................2

Number of times _________

Refused ………………………………………………………………………...3

Don't know………………………………………………………………………4

**Now I have some questions that ask about smoking.**

1. **Do you currently smoke cigarettes?**

YES 1

NO 2 **(SKIP TO 32)**

DON’T KNOW 3  **(SKIP TO 32)**

Refusal ……… …………………………………………………. 4  **(SKIP TO 32)**

Not Applicable …… …………………………………………….. 5  **(SKIP TO 32)**

1. **IF yes, how many cigarettes per day?**

< 10 per day (half a pack)……………… ……………………..1

10-20 per day (about a pack)…………………………………...2

More than a pack ………………………………………………..3

Don’t know …………………………………………………….4

Refusal …………………………………………………………. 5

Not Applicable …………………………………………………. 6

**If she did not self-sample, skip to 35.**

**We also want to know how competent you felt in your ability to self-sample as part of this study, regardless of whether you chose to self-sample or not. The following questions address how you feel about self-sampling.**

1. **Did you feel confident in your ability to self-sample?**

Not at all……………………………………………………………………………….1

Somewhat ……………………………………………………………………….……4

Definitely…………………………………………………..…………………...………7

1. **Did you feel capable of self-sampling?**

Not at all……………………………………………………………………………….1

Somewhat …………………………………………………………………….………4

Definitely………………………………………………………………………………7

1. **Did you believe you were able to self-sample correctly?**

Not at all……………………………………………………………………………….1

Somewhat ……………………………………………………………………………4

Definitely………………………………………………………………………………7

1. **Do you know what the self-sampler is testing for?**

Not at all……………………………………………………………………………….1

Somewhat ……………………………………………………………………………4

Definitely……………………………………………………………………...………7

1. **Do you understand why screening for HPV is important to prevent cervical cancer?**

Not at all……………………………………………………………………………….1

Somewhat ……………………………………………………………………………4

Definitely………………………………………………………………………………7

1. **Do you understand the connection between HPV and cervical cancer?**

Not at all……………………………………………………………………………….1

Somewhat ………………………………………………………………………….…4

Definitely…………………………………………………….…………………………7

1. **Do you know what to do if you receive positive HPV results?**

Not at all……………………………………………………………………………….1

Somewhat ……………………………………………………………….……………4

Definitely……………………………………………………………………………….7

**The next questions ask about your experiences with your CHW.**

1. **Do you feel that your CHW has provided you with choices and options about staying healthy?**

Not at all……………………………………………………………………………….1

Somewhat …………………………………………………………………………….4

Definitely………………………………………………………………………………7

1. **Do you feel your CHW understands how you see things with respect to your health?**

Not at all……………………………………………………………………………….1

Somewhat ……………………………………………………………………….……4

Definitely………………………………………………………………………………7

1. **Do you feel your CHW conveys confidence in your ability to make changes regarding your health?**

Not at all……………………………………………………………………………….1

Somewhat ……………………………………………………………………………4

Definitely………………………………………………………………………………7

1. **Do you feel your CHW listens to how you would like to do things regarding your health?**

Not at all……………………………………………………………………………….1

Somewhat ……………………………………………………………………………4

Definitely………………………………………………………………………………7

1. **Do you feel your CHW encourages you to ask questions about your health?**

Not at all……………………………………………………………………………….1

Somewhat ……………………………………………………………………………4

Definitely………………………………………………………………………………7

1. **Do you feel your CHW tries to understand how you see your health before suggesting any changes?**

Not at all……………………………………………………………………………….1

Somewhat ……………………………………………………………………………4

Definitely………………………………………………………………………….……7

1. **Do you feel your CHW tries to understand how your cultural values relate to your health?**

Not at all……………………………………………………………………………….1

Somewhat ………………………………………………………………………….…4

Definitely…………………………………………………………………………….…7

1. **Do you feel your CHW has been respectful of your beliefs and traditions as they relate to your health?**

Not at all……………………………………………………………………………….1

Somewhat ……………………………………………………………………………4

Definitely…………………………………………………………………………….…7

**The next set of questions also ask you about your Community Health Worker (CHW).**

**Using a scale of 1 to 5 where 1 is very poor/not at all and 5 is very good/very much, how would you rate the following characteristics of your assigned CHW?**

**1= very poor/ not at all**

2= poor / not very much

**3= fair/ half and half**

4= good / some

**5= excellent / very much**

1. Her friendliness/ courtesy 1 2 3 4 5
2. She talked to you using words you could understand 1 2 3 4 5
3. Her concern for your questions or worries 1 2 3 4 5
4. The helpfulness of information she provided for you 1 2 3 4 5
5. Her concern for your privacy and confidentiality of the

information you provided to her 1 2 3 4 5

1. Convenience of time for meetings and sessions she held with you 1 2 3 4 5
2. The amount of time she spent with you 1 2 3 4 5
3. Your overall confidence and trust in this CHW 1 2 3 4 5
4. Likelihood you would recommend this CHW

to another woman needing a PAP 1 2 3 4 5

**In addition to getting a PAP smear the CHWs also helped some of our participants in other items of concern. In the next set of questions we will ask you if the CHW helped you with some of these other items. Please be aware that some or all of these may not apply to you.** (yes or no)

1. Did your CHW help you in trying to get health insurance for you? Y / N
2. Did your CHW help you in trying to get health insurance for anyone

else in your family including your children? Y / N

1. Aside from a Pap smear, did your CHW help you in trying to obtain

other medical services for you? (examples include appointments

for routine physical exams, medical problems, vaccinations

or dental problems) Y / N

1. Did your CHW help you in trying to obtain other medical services

for others in your family including your children? Y / N

1. Did your CHW help you obtain assistance for any problems related

to mental or emotional health? Y / N

1. Did CHW help you get low cost medical services or connect you with

medical care at ______ *(insert* here as appropriate *Citrus, CHI, CHS*)? Y / N

1. Did your CHW help you obtain assistance for any other social services?

Examples of these programs include food stamps, employment assistance,

housing assistance, refugee assistance, after-earthquake assistance

programs, and others like these? Y / N

1. Did your CHW help you obtain assistance on other social services for

any members of your family? Y / N

1. If money was not a barrier, would you have had your PAP test

as recommended? Y / N

1. Did your CHW refer you to participate in any other research studies? Y / N
2. Did the CHW help with anything else we have not covered? Y / N

If YES, with what specifically?_______________________ ________

The following questions will ask about your experience using the self-sampler, depending on whether you were mailed the self-sampler or whether the CHW provided you with the self-sampler in person.

1. Did you receive the self-sampler in the mail?

YES 1 **(SKIP TO 71)**

NO 2

1. Did the CHW give you good directions on how and why to use the self-sampler?

YES 1

NO 2

1. Which of the following would have made understanding the CHW’s directions easier (circle all that apply)?

A more detailed explanation………………………………………………………………….1

More pictures. If yes, what specific pictures would you have liked?

_____________________________________________________..............................2

A video ………………………………………………………………………………………...3

A more graphic explanation of the instructions …………………………………………….4

Other, please specify_____________________________________

______________________________________________________

______________________________________________________……………..…….5

1. How did you feel about detail of the directions the CHW gave you? Were they too little, too much, or just enough?

Just enough ………. 1

Too Little ………………..2

Too Much ……………….3

1. Which of the following would have made understanding the directions easier (circle all that apply)?

A more detailed explanation………………………………………………………………….1

More pictures. If yes, what specific pictures would you have liked?

_____________________________________________________..............................2

A video ………………………………………………………………………………………...3

A more graphic explanation of the instructions …………………………………………….4

Other, please specify_____________________________________

______________________________________________________

______________________________________________________……………..…….5

1. How did you feel about the quantity of directions provided in the envelope? Was there too little, too much, or just enough?

Just enough ………. 1

Too Little ………………..2

Too Much ……………….3

1. Were you nervous that you might perform the test incorrectly?

YES 1

NO 2

1. Do you think you performed the test correctly?

YES 1

NO 2

1. If you had a choice would your prefer to perform the test at home or at a clinic?

At home ………………..1 **(SKIP TO 77)**

At a clinic ………………2

1. If at the clinic, which of the following is the reason why:

A physician could guide me if I had trouble ………………………………1

I would feel more comfortable in a medical surrounding ………………..2

A doctor or nurse could perform the test on me ………………………….3

Other__________________________________ .................................4

1. If at home, which of the following is the reason why:

It is more convenient ………………………………………………………..1

I feel more freedom at home ……………………………………………….2

I would be embarrassed at a clinic ………………………………………...3

Other__________________________________ ………………………..4

1. If the choice was to perform the test at home or not to do the test at all which would you choose?

Perform the test at home ……………………………………………………1

I would not perform the test ..……………………………………………….2

1. What do you think could prevent a woman from performing the test (circle all that apply)?

She will not perform the test because she does not think she is ill …………………….1

She doesn’t have time to perform the test ………...………………………………………2

She will not be able to read the directions ………………………………………………...3

Her husband will not want her to perform the test ………………………………………..4

She will not do the test because she is afraid of the results …………………………….5

Other:_______________________________________________________

____________________________________________________________

____________________________________________________________ …………6

***Exit script:*  This was the last of the questions. Thank you for your time!**

**End Time:** _____________  **Survey completed:** Yes ____ No_____

**Reasons why not: *_____________________________________________***

**Any other notes:** _______________________________________
