# Supplementary figures and images for: Mailed HPV self-sampling for cervical cancer screening among underserved minority women: study protocol for a randomized controlled trial
Source: Trials. 2017 Jan 13;18:19. doi: 10.1186/s13063-016-1721-6 (PMC5237204; doi:10.1186/s13063-016-1721-6)

Trial Flow Diagram


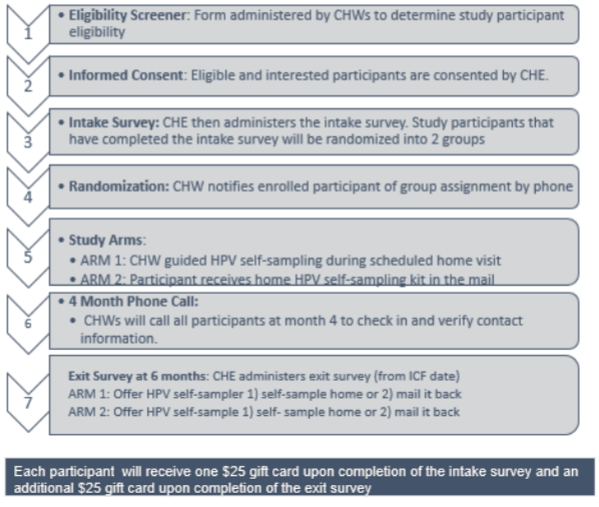

Supplement: Additional file 5: — Trial flow diagram. (DOCX 153 kb) [file 13063_2016_1721_MOESM5_ESM.docx]
